# Comparison of sheep and human middle-ear ossicles: anatomy and inertial properties

**DOI:** 10.1007/s00359-020-01430-w

**Published:** 2020-06-20

**Authors:** Dominik Péus, Ivo Dobrev, Flurin Pfiffner, Jae Hoon Sim

**Affiliations:** 1 University Hospital Basel, Basel, Switzerland; 2 Department of Otorhinolaryngology, Head and Neck, Surgery University Hospital Zurich, Zurich, Switzerland; 3 University of Zurich, Zurich, Switzerland

**Keywords:** Hinge-like rotational motion, Middle-ear ossicles, Moment of inertia, Principal moment of inertia, Sheep

## Abstract

The sheep middle ear has been used in training to prepare physicians to perform surgeries and to test new ways of surgical access. This study aimed to (1) collect anatomical data and inertial properties of the sheep middle-ear ossicles and (2) explore effects of these features on sound transmission, in comparison to those of the human. Characteristic dimensions and inertial properties of the middle-ear ossicles of White-Alpine sheep (*n* = 11) were measured from high-resolution micro-CT data, and were assessed in comparison with the corresponding values of the human middle ear. The sheep middle-ear ossicles differed from those of human in several ways: anteroinferior orientation of the malleus handle, relatively small size of the incus with a relatively short distance to the lenticular process, a large area of the articular surfaces at the incudostapedial joint, and a relatively small moment of inertia along the anterior–posterior axis. Analysis in this study suggests that structure and orientation of the middle-ear ossicles in the sheep are conducive to an increase in the hinge-like ossicular-lever-action around the anterior–posterior axis. Considering the substantial anatomical differences, outcomes of middle-ear surgeries would presumably be difficult to assess from experiments using the sheep middle ear.

## Introduction

Various animal models have been used for both basic science and clinical hearing research. For basic hearing science, the aims are mainly to reveal the hearing capacity of the targeted animal or to perform experiments to reveal hearing mechanisms, which are generally difficult with live human subjects. For clinical purposes, animal models can be used for training of various surgeries or for tests of new surgical techniques and devices in developmental stages.

Small animals are widely used for these research purposes, most common being rats and mice, as well as other small vertebrate animals such as rodents (e.g., gerbils, chinchilla, and guinea pigs), rabbits, and cats. However, small animals are different from humans anatomically and physiologically (e.g., difference in shape and size of the middle ear shown in Hemila et al., 1995 and Nummela, 1995); therefore, the findings can differ widely. Such limitations become more significant when the animal models are used as surrogates of human beings for clinical purposes. With an expectation to reflect the anatomy of the human ear more adequately, large animal models have been evaluated as well. For example, the pig has been evaluated as a possible animal model, but the soft and fatty tissues overlying the mastoid make experimental approaches difficult (Schnabl et al. 2012). Primates, being closer to human phylogenetically as well in size, can be an alternative, but they are prohibited for use in experiments except for exceptional cases.

The sheep has been considered as an animal model because it can be obtained easily and the size of the ear is similar to that of the human ear. According to studies by Seibel et al. (2006a, b), the average size of the middle and inner ears of the sheep is approximately two-thirds the average size of the middle and inner ears of the human. Thereby, the sheep has been used for research on bone conduction (Gerhardt et al. 1996; McFadde et al. 2008), training of middle- and inner ear surgeries (Gocer et al. 2007; Cordero et al. 2011; Mantokoudis et al. 2015), and assessment of new surgical techniques and hearing devices (Lavinsky et al. 1999; Neudert et al. 2010; Miller et al. 2014; Larsson et al. 2015; Pfiffner et al. 2018).

**Fig. 1:**
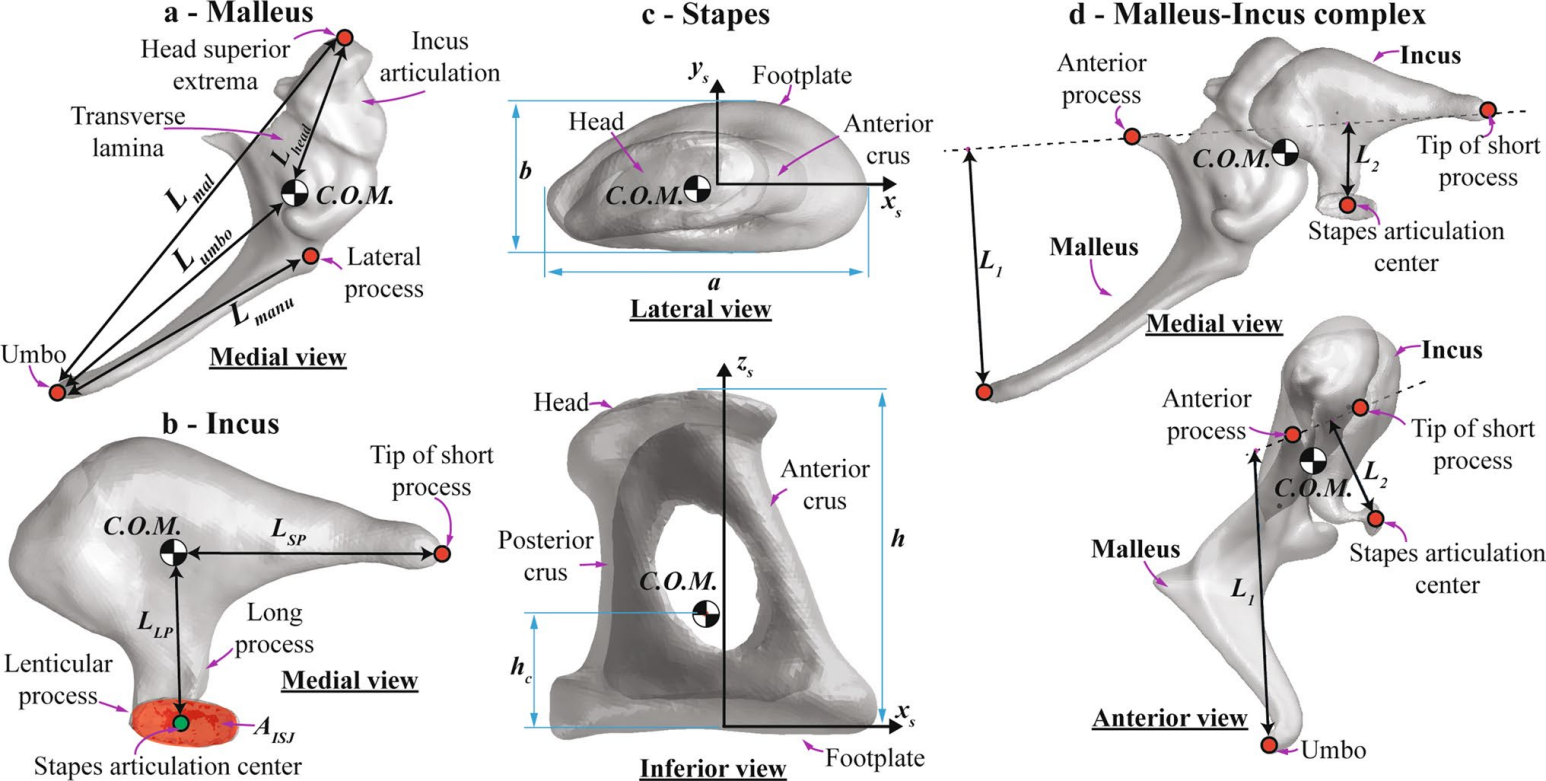
References for dimensions of the middle-ear bones of sheep (right ear). Characteristic lengths of the malleus (**a**), characteristic lengths and area of the interface to the stapes head by the incus (**b**), characteristic lengths of the stapes (**c**), and lever ratio of the hinge-like rotational motion of the malleus–incus complex (**d**). The checkered circles indicate the center of mass of the malleus, incus, stapes, and malleus–incus complex, and dashed lines in d represent the presumed position of the hinge-like rotational axis. *L*_1_ and *L*_2_ in d represent 3D distances from the rotational axis to the tip of the umbo and the centroid of the articular surface of the incus lenticular process to the stapes head, respectively

Although the sheep ear has been considered as a suitable animal model, comprehensive anatomical and biomechanical data and comparisons with the human middle ear do not exist. For such comparisons, biomechanical models of the sheep middle ear are required but have not been established due to lack of data. According to Péus et al. (2017), the magnitude ratio of the velocity at the lenticular process of the incus (the ovoid bony part attached to the distal end of the long process of the incus; see Fig. 1) or stapes relative to the velocity at the umbo in the sheep is appreciably smaller than the corresponding magnitude ratio in the human (Dobrev et al. 2016), indicating that middle-ear mechanics in the sheep may be different from middle-ear mechanics in the human. Several studies have been done to document the anatomy of the sheep middle ear (Seibel et al. 2006b; Gurr et al. 2011); however, these studies have not provided sufficient quantitative information for mathematical formulation of a model.

This study aims to provide and assess quantitative information on the sheep middle-ear ossicles using micro-CT images to determine characteristic lengths, sizes, and inertial properties based on three-dimensional (3D) reconstruction and to compare these outcomes with corresponding human data. Based upon these data, possible effects on mechanics of the sheep middle-ear ossicular chain will be proposed.

## Materials and methods

### Collection and micro-CT imaging of sheep middle-ear bones

Ossicular structures were dissected from the post-mortem temporal bones of White-Alpine sheep (Rizzi 2009). The age of the sheep was not noted, but they were generally within a range of 1–2 years old. The total sample of ossicular bones consisted of five mallei, 11 incudes and eight stapedes. Of these, there was one malleus–incus complex and an intact middle-ear chain. Samples for the three different bones were not equal because of breakage either during dissection or handling.

The middle-ear ossicles were imaged using a *μCT 40* micro-CT machine (SCANCO Medical AG, Switzerland) with an isotropic voxel size of 6, 8 or 10 μm (depending on available time slots of the CT machine) for the isolated malleus, incus, and stapes, and with isotropic voxel sizes of 10 μm and 15 μm for the malleus–incus complex and intact middle-ear chain, respectively. The X-ray intensity and tube voltage were set to 145 μA and 55 keV, respectively (Sim et al. 2007, 2013; Sim and Puria 2008). The number of projections per 180 degrees was set to 1000, and the integration time (exposure time per image) was set to 380 ms.

The micro-CT images were segmented for 3-D volume reconstruction of the bones using the Evaluation Program (SCANCO Medical AG, Switzerland). The segmentation was performed in two steps. First, the bones were separated from the ambient structures or air by an outline contour in each slice image. The outline contours were made by a combination of hand drawing and a “shrink-wrapping” algorithm in the Evaluation Program (SCANCO Medical AG, Switzerland). Next, selection of a specific range of the grayscale was executed. The selected grayscale range was from 250 to 1000 in the Evaluation Program, where the grayscale levels 0 and 1000 corresponded to no attenuation (*μ*/*ρ* = 0) and the maximum attenuation (*μ*/*ρ* = 8 cm^2^/g), respectively (Sim et al. 2007; Sim and Puria 2008). The 3D volume data of the middle-ear bones were generated from the segmentation, and these data, which contained a non-zero value for the bony parts and a zero value for the other parts, were stored as a file format of AIM, a specific binary format for the Evaluation Program. In addition, the surface models of the 3D volumes were generated in a file format of Standard Tessellation Language (STL). Additional details of the procedures are available in our previous publications (Sim et al. 2007, 2013; Sim and Puria 2008).

### Measurement and characterization of dimensions

Anatomical dimensions of the middle-ear ossicles were measured using the built-in functions in *RapidForm XOS2* (3D Systems Corp., Korea). The reference points, surfaces, axis, and frames except for the center of mass (C.O.M.) were extracted from the STL surface models of the 3D volumes, and characteristic lengths and areas were measured based on the references. The volumes of the ossicles were also measured from the STL surface models. The C.O.M. was obtained from the 3D volume data of the AIM format using custom-made Matlab (The MathWorks Inc., USA) codes (see “Calculation of inertial properties”).

Considering analogy of orientation of the middle-ear ossicles between the human and the sheep, the bipedal-centric terms of “anterior–posterior”, “superior–inferior”, and “lateral–medial”, which refer to the orientations of the middle ear in the human skull, are used for indicating anatomical directions of the middle ear in this article. While the anterior and posterior directions correspond to the dorsal and ventral directions in the human, they correspond to the cranial and caudal directions in the sheep. Similarly, the superior and inferior directions correspond to the cranial and caudal directions in the human whereas they correspond to the dorsal and ventral directions in the sheep. Use of the bipedal-centric terms for anatomical directions of the middle ear is justified by the fact that that the line drawn through the zygomatic arch and the center of the bony porus of the external ear canal, which is used to define a horizontal reference line of middle-ear orientation, is oriented along the anterior–posterior direction in both species. We have also seen that the tip of the manubrium of the malleus points in both species in an inferior direction relative to the reference line (zygomatic arch and center of the bony porus of the external ear canal). Since isolated ossicles were used in this study, orientation of the isolated ossicles relative to the head could not be identified. The anatomical orientations of the isolated ossicles in this article were defined based on orientation of the stapes footplate, assuming that the long and short axes of the stapes footplate are aligned along the anterior–posterior direction and the superior direction, respectively. With such definition of the anatomical directions, orientation of the incus was similar in both species. The long process of the incus is aligned approximately along the superior–inferior direction in both species. The incus is located posteriorly to the malleus, and the stapes is located medially to the incus.

For the malleus (Fig. 1a), the extracted references included the C.O.M., the tip (most superior point) of the malleus head, the tip (inferior extrema point) of the umbo (the bottom end of the malleus handle; connected to the center of the tympanic membrane), and the tip (most lateral point) of the lateral process (conical projection at the lateral end of the malleus handle; attached to the upper part of the tympanic membrane). Based on these reference points, the following characteristic lengths were defined: the total length (*L*_*mal*_) defined as the distance from malleus head tip to the umbo tip, the manubrium length (*L*_*manu*_) defined as the distance from the lateral process tip to the umbo tip, and the distances between the malleus head tip (*L*_*head*_) and the umbo tip (*L*_*umbo*_) to the C.O.M. of the malleus.

For the incus (Fig. 1b), the extracted references included the C.O.M., the tip of the short process, the articular surface of the lenticular process to the stapes, and the centroid of the articular surface (calculated from the 3D surface). Based on these reference points and area, the following characteristic parameters were defined: the lengths of the short (*L*_*SP*_) and long processes (*L*_*LP*_) defined as the distances from the C.O.M. of the incus to the tip of the short process and the centroid of the articular surface, respectively; the area of the articular surface of the lenticular process (*A*_*ISJ*_). The terms “long” and “short” processes of the incus are based on anatomy of the human incus, and the short process is actually the longer of the two in the sheep.

For measurement of the stapes dimensions (Fig. 1c), the C.O.M., the medial surface of the stapes footplate, the centroid of the medial surface of the stapes footplate (calculated from the 3D surface), the lateral surface of the stapes head, and the centroid of the lateral surface (calculated from the 3D surface) were extracted as references. Then, a local anatomical frame based on the medial surface of the stapes footplate was created (Sim et al. 2012, 2013), and was applied to each of the eight samples. The local anatomical frame of the stapes was made such that the *x*_*S*_-axis and *y*_*S*_-axis are along the long (aligned along the anterior–posterior direction) and short (aligned approximately along the superior–inferior direction) axes of the stapes footplate, respectively, and the origin was located on the centroid of the medial surface. Consequently, the line perpendicular to footplate (aligned approximately along the lateral-medial direction) was set as the *z*_*S*_-axis. The positive *x*_*S*_-, *y*_*S*_-, and *z*_*S*_-directions were set toward the anterior, superior, and lateral directions, respectively. Such alignment of the *x*_*S*_*-*, *y*_*S*_*-*, and *z*_*S*_*-*axes resulted in a right-handed frame system for right ears and a left-handed frame system for left ears. Since the medial surface of the stapes footplate interfaces cochlear fluid, motions of the stapes footplate (motions of the medial surface of the stapes footplate more exactly) become stimuli to cochlear activation. Physiological motions of the stapes footplate in 3D space are decomposed into three elementary motion components of translation motion along a direction perpendicular to the footplate (called piston-like motion component), rotational motions along the long and short axes of the footplate (called rocking-like motion components) (Békésy 1960; Kirikae 1960; Heiland et al. 1999; Huber et al. 2001; Hato et al. 2003; Decraemer et al. 2000, 2007; Ravicz et al. 2008; Sim et al. 2010). The local coordinate system based on the medial surface of the stapes footplate, and its long and short axes, is used to describe the elementary motion components of the stapes footplate and anatomy of the stapes with consideration of stimuli to cochlear activation. Based on these references and the coordinate system, the following quantities were defined and measured: the total height (*h*) of the stapes defined as the distance from the centroid of the footplate surface to the centroid of the stapes head surface; the height (*h*_c_) of the C.O.M. defined as the distance from the centroid of the footplate surface to the C.O.M.; the long (*a*) and short (*b*) lengths of the footplate; footplate area on the medial side based on the full area (*A*_*FT*_) of the corresponding 3D surface and the projected area on the *x*_*S*_*–y*_*S*_ plane (*A*_*FTproj*_) on a plane numerically fitted to the 3D surface (Sim et al. 2013).

In addition to the references defined for the isolated malleus, incus, and stapes, the coordinates of the C.O.M. of each ossicle of the malleus, incus, and stapes in a global anatomical frame and the rotational axis of the hinge-like motion of the malleus–incus complex were extracted for the one sample of the entire middle-ear ossicular chain. The global anatomical frame of the *xyz* coordinate system was obtained such that the positive *x*-, *y*-, and *z*-directions were the same as the positive *x*_*S*_-, *y*_*S*_-, and *z*_*S*_-directions and the origin was located on the C.O.M. of the entire ossicular chain (Dobrev et al. 2016). The rotational axis of the hinge-like motion of the malleus–incus complex was assumed to be along a line passing through the anterior process of the malleus and the tip of the short process of the incus, where the ossicles are physically tethered to the skull. It is presumed that at least at low frequencies, the malleus–incus complex in mammals including the human shows hinge-like rotational motion about the rotational axis (Dahmann 1929; Wever and Lawrence 1954; Webster 1961; Dallos 1973; Manley and Johnson 1974; Fleischer 1978; Lavender et al. 2011; Mason 2001, 2016). Measurements of three-dimensional motion of the malleus–incus complex by Decraemer et al. (2014) showed that the rotational axis in the gerbil is close to the rotational axis defined above at low frequencies below a few kilohertz. Three-dimensional motion of the sheep middle-ear ossicular chain has not been measured, but it was assumed that the sheep middle ear has such a rotational motion along the rotational axis defined above at low frequencies as well. With the hinge-like rotational motion along the rotational axis, the arm lengths *L*_*1*_ and *L*_*2*_ of the rotational motion were determined as the 3D distances from the rotational axis to the tip of the umbo and the centroid of the articular surface of the incus lenticular process to the stapes head, respectively (Fig. 1d). The lever ratio of the malleus–incus complex (*L*_*1*_/*L*_*2*_) determines the magnitude ratio of motion of the lenticular process of the incus to motion of the umbo.

### Calculation of inertial properties

Moments of inertia of a rigid body in rotational motions act like mass in translational motions (i.e., when the external excitation for rotational motion is the same, rotational motion of a smaller magnitude is generated with a larger moment of inertia). The moment of inertia *I* along a rotational axis is calculated by

1 $$I = \smallint r^{2} dm,$$

where *r* indicates the distance of the infinitesimal mass *dm* from the rotational axis. Since the moment of inertia of a rigid body along a rotational axis is determined by mass distribution with respect to the rotational axis, the moment of inertia can be different for rigid bodies with the same mass and the same rotational axis. While mass in translational motion of a rigid body is isotropic independently of the direction of translational motions, the moment of the inertia of a rigid body varies with a rotational axis. The principal axes of a rigid body are defined as the three orthogonal axes of a reference frame when non-diagonal terms of the inertial matrix in the reference frame become zero (i.e., *I*_*xy*_ = *I*_*yz*_ = *I*_*zx*_ = 0 in Sim et al. 2007). The three moments of inertia along the three principal axes are called principal moments of inertia. The three principal axes pass through the C.O.M. of the rigid body, and the moments of inertia of the rigid body becomes maximum and the minimum along the two of the three principal axes. The moment of inertia along a rotational axis becomes larger and smaller when orientation of the rotational axis becomes closer to the principal axis with the maximum principal moment of inertia and the principal axis with the minimum principal moment of inertia, respectively. Difference between the three principal moments of inertia indicates how sensitive the moment of inertia is with change of the rotational axis. Once the principal axes and the corresponding principal moments of a rigid body are obtained, the moment of inertia of the rigid body along any rotational axis can be calculated by frame transformation of the principal inertia matrix. When a rigid body has a moment of inertia *I*_*COM*_ along an axis passing through the C.O.M. of the rigid body, a new moment of inertia *I* along a new axis, which does not pass through the C.O.M. and is parallel to the first axis, can be calculated by

2 $$I = I_{COM} + md^{2} ,$$

where the *m* is mass of the rigid body, and *d* is the distance between the two axes. Equation (2) indicates that the moment of inertia becomes larger as the distance between the rotational axis and the C.O.M. of the rigid body becomes larger.

The C.O.M., principal axes, and corresponding principal moments of inertia of the three middle-ear bones were calculated from the 3D volume data in AIM file format, using custom-made Matlab codes. The AIM file contains data of distribution of voxels of a non-zero value (i.e., voxels corresponding to bony parts) in 3D space. All of the formulae necessary for the calculation with the standard discretization are available in Sim et al. (2007). In the work by Sim et al., the low-density parts inside the ossicle were considered for calculation of the inertia properties. According to the work, the low-density parts observed inside the middle-ear ossicles have grayscale levels distinguishable from those of the air and bony parts in micro-CT images. Since the grayscale values of the low-density parts correspond to the grayscale values of the soft tissues and fluids (Sim et al. 2007, 2008), the low-density parts inside the ossicle are considered to be associated with blood vessels, marrow spaces, or fluids. They are classified into two categories; (1) the low-density parts distributed widely inside the ossicle, and (2) the low-density parts establishing a large hollow space inside the ossicle. The first case is observed in the malleus head and the incus body of the human, and volume fraction of the low-density parts occupy 3–14% of the entire volume of the malleus and incus (human) and an even lower volume in the stapes (Sim et al. 2007, 2013). The second case is observed in the malleus handle of the cat, which is hollow along the center line (Puria and Steele 2010). The low-density parts are clearly visible in the slice images for both cases. Such low-density parts were not visible in micro-CT images obtained for the sheep osscles in this study, and the volume of the ossicle with consideration of only the high-density parts (i.e., bony parts) reaches 98–99% of the total ossicular volume calculated from the surface model in STL format. The low-density parts were ignored in the calculations (i.e., zero mass was assigned to the low-density parts and mass of the bone was assigned only into the high-density parts) because it was assumed that the low-density parts have a relatively small contribution to the inertial properties. The effects of the density *ρ*_*L*_ of the low-density parts on calculation of the density *ρ*_*H*_ of the high-density parts can be examined by the following formula (modification of Eq. (6) in Sim et al. 2007).

3 $$\rho_{H} = \frac{{m - v_{L} \rho_{L} V}}{{\left( {1 - v_{L} } \right)V}},$$

where *m* and *V* represent the mass and volume of the ossicle, and *v*_*L*_ the volume fraction of the low-density parts. With the mass and volume of the sheep middle-ear ossicles (Table 5), the density of the high-density parts with *ρ*_*L*_ = 0 becomes larger than the density with *ρ*_*L*_ = 1 mg/mm^3^ (density of water), by a factor of 0.0045 (0.45%) when the volume fraction of the low-density parts (*v*_*L*_) is 1% and by a factor of 0.0091 (0.91%) when the volume fraction of the low-density parts is 2%.

The mass of each middle-ear ossicle was averaged from measurements with two or three bones using a scale of ME 204 T (Mettler-Toledo LLC, USA). Then the average density of each middle-ear ossicle was calculated from the average mass and average volume. The average densities were applied to calculation of the inertial values.

### Human reference data

The human reference data, which were necessary for comparison with the corresponding sheep data, were obtained in part from our previous research (inertial properties of the malleus, incus, and malleus–incus complex from Sim et al. 2007, and dimensions and inertial properties of the stapes from Sim et al. 2013). As the characteristic lengths of the malleus and incus were not available in the literature, eight mallei and eight incudes were scanned by micro-CT, their 3D volumes reconstructed, and the corresponding characteristic lengths measured. Additionally, one intact human middle-ear ossicular chain was scanned by micro-CT to obtain the moment of inertia of the entire ossicular chain.

Conditions for micro-CT imaging and segmentation of the 3D volumes, and methods applied to obtain the characteristic dimensions and inertial properties were the same for the human and sheep samples, except for the methods used for calculation of inertial properties of the human malleus and incus. While the density of the water was assigned to the low-density parts associated with the blood vessels in the human reference data of the inertial properties of the malleus and incus from Sim et al. (2007), zero density was assigned to the low-density parts in this study. However, with the small portion of the low-density part considered, the differences are presumed to be small, as described in “(Calculation of inertial properties)”.

## Results

### Dimensions and inertial properties of the malleus

Table 1 lists the dimensions and inertial properties of the sheep malleus in comparison with the corresponding values of the human malleus. While the characteristic lengths of the sheep malleus were similar to the corresponding lengths of the human malleus, the volume of the human malleus was more than twice the volume of the sheep malleus, indicating that the sheep malleus is slenderer than the human malleus. The ratios of the volume and mass of the malleus relative to the volume and mass of the total ossicular chain (*V*^*M*^/*V*^*MIS*^ and *m*^*M*^/*m*^*MIS*^) were larger in the sheep. The ratios of the two characteristic lengths from the center of mass (*L*_*head*_ and *L*_*umbo*_) were similar between the sheep and human, indicating that the relative locations of the center of mass are similar.

**Table 1:** Dimensions and inertial properties of the sheep malleus in comparison with the human malleus

Description	Symbol	^1^Sheep	^2^Ref. human	Sheep/human
Length from top of head to umbo	*L*_*mal*_ (mm)	7.88 ± 0.51	8.15 ± 0.37	0.97
Length of manubrium	*L*_*manu*_ (mm)	5.26 ± 0.39	4.78 ± 0.30	1.10
Ratio of *L*_*manu*_–*L*_*mal*_	*L*_*manu*_/*L*_*mal*_	0.67 ± 0.02	0.59 ± 0.03	1.13
Length from C.O.M. to top of head	*L*_*head*_ (mm)	2.96 ± 0.22	2.75 ± 0.29	1.08
Length from C.O.M. to umbo	*L*_*umbo*_ (mm)	5.32 ± 0.27	5.52 ± 0.22	0.96
Ratio of *L*_*head*_–*L*_*umbo*_	*L*_*head*_/*L*_*umbo*_	0.56 ± 0.01	0.50 ± 0.05	1.12
Volume	*V*^*M*^ (mm^3^)	5.75 ± 0.65	12.7 ± 2.0	0.45
Mass	*m*^*M*^ (mg)	13.4	30.3 ± 4.6	0.44
Mass ratio relative to total ossicular chain	*m*^*M*^/*m*^*MIS*^	0.59	0.46	1.27
Average density	*ρ*^*M*^ (mg/ mm^3^)	2.33	2.39	0.97
Minimum principal moment of inertia	*I*^*M*^_*MIN*_ (mg·mm^2^)	7.8 ± 2.7	17.3 ± 4.0	0.45
Medium principal moment of inertia	*I*^*M*^_*MED*_ (mg·mm^2^)	41.1 ± 10.6	100.6 ± 17.6	0.41
Maximum principal moment of inertia	*I*^*M*^_*MAX*_ (mg·mm^2^)	45.5 ± 12.3	106.1 ± 18.9	0.43
Ratio of *I*^*M*^_*MIN*_–*I*^*M*^_*MAX*_	*I*^*M*^_*MIN*_/*I*^*M*^_*MAX*_	0.17 ± 0.02	0.16 ± 0.01	1.06
Ratio of *I*^*M*^_*MED*_–*I*^*M*^_*MAX*_	*I*^*M*^_*MED*_/*I*^*M*^_*MAX*_	0.91 ± 0.02	0.95 ± 0.00	0.96

^**1**^*n* = 5 except for mass and density (*n* = 2 for mass; the average density was obtained from mean values of volume and mass)

^**2**^*n* = 8 for lengths, and from (Sim et al. 2007) for volume, mass, and average density, and principal moments of inertia

**Fig. 2:**
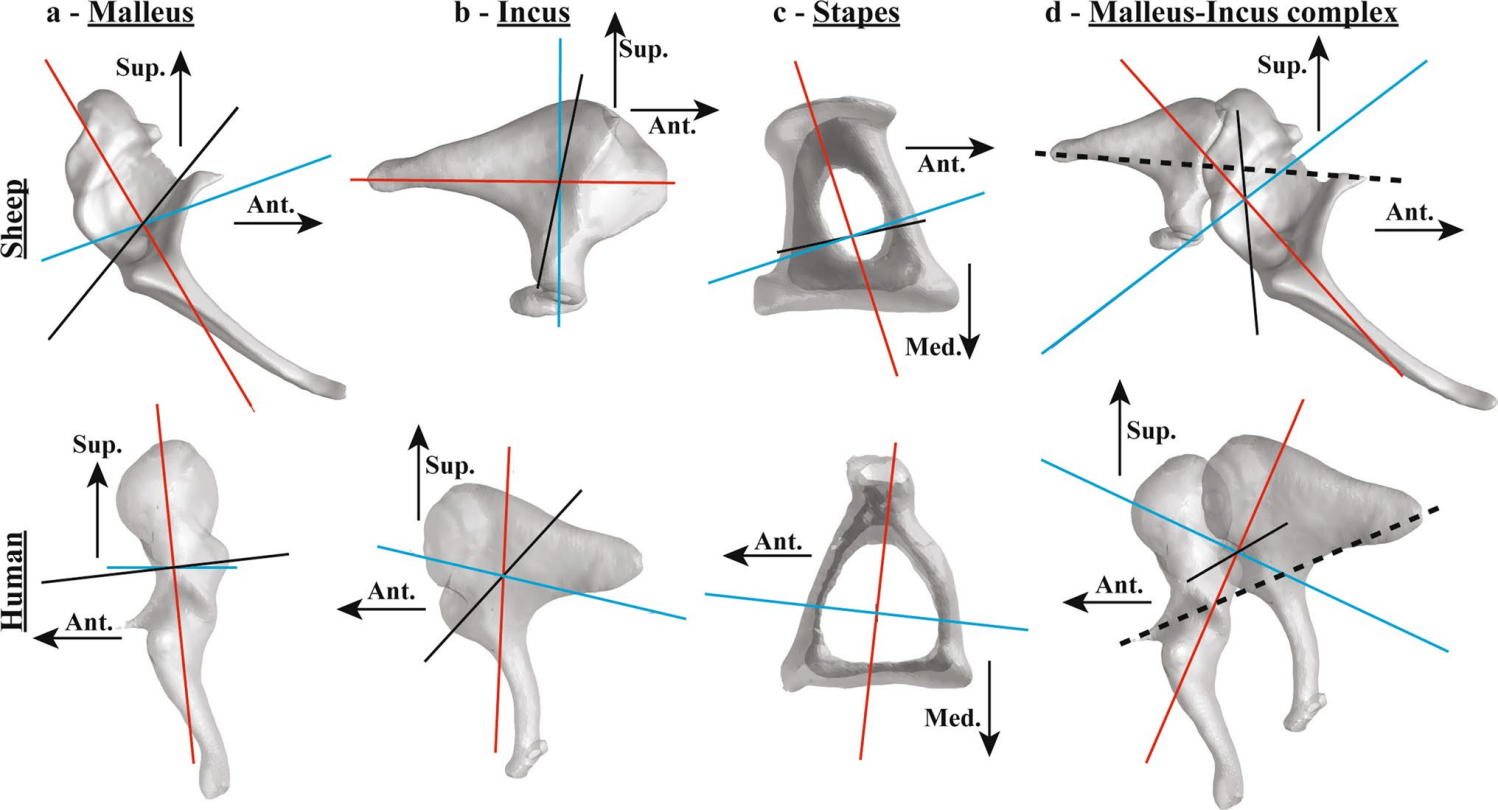
Principal axes of the sheep (right ear) and human (left ear) middle-ear ossicles: Malleus (**a**), incus (**b**), stapes (**c**), and malleus–incus complex (**d**). The red, blue, and black lines indicate the principal axes with the minimum, medium, and maximum principal moments of inertia, respectively. The intersection of the principal axes in each figure corresponds to the ossicular center of the mass (C.O.M.), and dashed lines in d represent the presumed position of the hinge-like rotational axis

While the principal moments of inertia of the human malleus were more than twice those of the sheep malleus, the relative ratios between the three principal moments of inertia were similar. The principal axes of the malleus in the sheep and human are shown in (Fig. 2a). The principal axis with the minimum moment of inertia (red line) is aligned approximately along the longitudinal direction of the malleus handle for both sheep and human. The principal axis with the maximum moment of inertia (black line) is aligned approximately along the anterior–posterior direction (approximately 20-degree deviation) in the human malleus, but is largely deviated from the anterior–posterior direction (66°) in the sheep malleus. The principal axis with the maximum moment of inertia in the sheep is closer to the lateral–medial direction (38°).

### Dimensions and inertial properties of the incus

Dimensions and inertial properties of the sheep incus in comparison with the corresponding values of the human incus are shown in (Table 2). The mass and volume of the sheep incus was only a quarter (24–26%) of the human incus. The mass ratio of the incus relative to the total ossicular chain (*m*^*I*^/*m*^*MIS*^) was almost 50% in the human, but only 34% in the sheep. The shapes of the sheep and human incus were different. The prominent difference was the relatively short length of the long process (*L*_*LP*_), and the relatively large area of the interface to the incudostapedial joint (*A*_*ISJ*_), in the sheep incus. The ratio *L*_*SP*_*/L*_*LP*_ was only 0.78 ± 0.06 in the human incus, but was 1.52 ± 0.12 in the sheep incus.

**Table 2:** Dimensions and inertial properties of the sheep incus in comparison with the human incus

Description	Symbol	^1^Sheep	^2^Ref. human	Sheep/human
Length from C.O.M. to posterior end	*L*_*SP*_ (mm)	2.52 ± 0.09	3.38 ± 0.26	0.75
Length from C.O.M.to ISJ	*L*_*LP*_ (mm)	1.67 ± 0.11	4.33 ± 0.17	0.39
Ratio of *L*_*SP*_–*L*_*LP*_	*L*_*SP*_/*L*_*LP*_	1.52 ± 0.12	0.78 ± 0.06	1.95
Area of ISJ	*A*_*ISJ*_ (mm^2^)	0.42 ± 0.05	0.24 ± 0.07	1.75
Volume	*V*^*I*^ (mm^3^)	3.81 ± 0.37	14.8 ± 2.5	0.26
Mass	*m*^*I*^ (mg)	7.73	32.0 ± 5.9	0.24
Mass ratio relative to total ossicular chain	*m*^*I*^/*m*^*MIS*^	0.34	0.49	0.69
Average density	*ρ*^*I*^ (mg/ mm^3^)	2.03	2.15	0.94
Minimum principal moment of inertia	*I*^*I*^_*MIN*_ (mg·mm^2^)	3.61 ± 0.56	35.3 ± 11.9	0.10
Medium principal moment of inertia	*I*^*I*^_*MED*_ (mg·mm^2^)	4.54 ± 0.69	59.5 ± 12.1	0.08
Maximum principal moment of inertia	*I*^*I*^_*MAX*_ (mg·mm^2^)	6.48 ± 1.02	84.3 ± 21.2	0.08
Ratio of *I*^*I*^_*MIN*_–*I*^*I*^_*MAX*_	*I*^*I*^_*MIN*_/*I*^*I*^_*MAX*_	0.56 ± 0.03	0.41 ± 0.03	1.36
Ratio of *I*^*I*^_*MED*_–*I*^*I*^_*MAX*_	*I*^*I*^_*MED*_/*I*^*I*^_*MAX*_	0.70 ± 0.03	0.71 ± 0.03	0.99

^**1**^*n* = 11 except for mass and density (*n* = 3 for mass; the average density was calculated from mean values of volume and mass)

^**2**^*n* = 8 for lengths and area of ISJ, and from Sim et al. 2007 for volume, mass, and average density, and principal moments of inertia

The principal moments of inertia in the sheep incus were 8–10% of the values in the human incus. The principal axis for the maximum moment of inertia (black lines in Fig. 2b) was aligned closely to the lateral-medial direction for both sheep and human. The axis for the minimum moment of inertia (red lines in Fig. 2b) was aligned approximately along the anterior–posterior direction for the sheep and along the superior–inferior direction for the human.

### Dimensions and inertial properties of the stapes

The volume and mass of the sheep stapes were 55–56% of those of the human stapes (Table 3). The sizes of the sheep stapes footplate were 71–73% in length and 51% in area, of the sizes of the human stapes footplate. The ratio between the long and short lengths of the footplate was similar for the sheep and human (*a*/*b* ≈ 2.2). The height of the center of mass relative to the total stapes height was almost the same for the sheep and human (*h*_*c*_*/h* ≈ 0.37), indicating the relative position of the center of mass is similar in both species. The ratio of the total height to the equivalent footplate diameter was larger in the human stapes, indicating that the human stapes has a relatively large footplate.

**Table 3:** Dimensions and inertia properties of the sheep stapes in comparison with the human stapes

Description	Symbol	^1^Sheep	^2^Ref. human	Sheep/human
Long length of footplate	*a* (mm)	2.03 ± 0.12	2.81 ± 0.16	0.72
Short length of footplate	*b* (mm)	0.93 ± 0.06	1.27 ± 0.11	0.73
Long length/short length of footplate	*a/b*	2.20 ± 0.19	2.22 ± 0.20	0.99
Footplate area (medial surface)	*A*_*FT*_ (mm^2^)	1.54 ± 0.14	3.03 ± 0.33	0.51
Projected footplate area (to *x*_*S*_*y*_*S*_-plane)	*A*_*FTproj*_ (mm^2^)	1.45 ± 0.13	2.86 ± 0.32	0.51
Projected footplate area/Footplate area	*A*_*FTproj*_*/A*_*FT*_	0.94 ± 0.01	0.94 ± 0.02	1.00
Equivalent diameter of footplate area	*d*_*FT_eq*_ (mm)	1.40 ± 0.06	1.96 ± 0.11	0.71
Equivalent diameter of projected footplate area	*d*_*FTproj_eq*_ (mm)	1.36 ± 0.06	1.90 ± 0.11	0.72
*x*-coordinate of the C.O.M	*x*_*c*_ (mm)	-0.12 ± 0.07	0.00 ± 0.09	
*y*-coordinate of the C.O.M	*y*_*c*_ (mm)	0.00 ± 0.04	−0.12 ± 0.09	
Height (*z*-coordinate) of the C.O.M	*h*_*c*_ (mm)	0.76 ± 0.05	1.22 ± 0.16	0.62
Total height of stapes	*h* (mm)	2.10 ± 0.10	3.28 ± 0.21	0.64
Height of C.O.M./total height	*h*_*c*_*/h*	0.37 ± 0.02	0.37 ± 0.04	1.00
Total height/equivalent diameter of footplate	*h/d*_*FTproj_eq*_	1.55 ± 0.11	1.72 ± 0.12	0.90
Volume	*V*^*S*^ (mm^3^)	0.83 ± 0.08	1.43 ± 0.24	0.56
Mass	*m*^*S*^ (mg)	1.75	3.2	0.55
Mass ratio relative to total ossicular chain	*m*^*S*^/*m*^*MIS*^	0.08	0.05	1.57
Average density	*ρ*^*S*^ (mg/ mm^3^)	2.11	2.24	0.94
Minimum principal moment of inertia	*I*^*S*^_*MIN*_ (mg·mm^2^)	0.49 ± 0.13	2.2 ± 0.49	0.22
Medium principal moment of inertia	*I*^*S*^_*MED*_ (mg·mm^2^)	1.05 ± 0.20	4.6 ± 1.18	0.23
Maximum principal moment of inertia	*I*^*S*^_*MAX*_ (mg·mm^2^)	1.37 ± 0.29	6.3 ± 1.53	0.22
Ratio of *I*^*S*^_*MIN*_–*I*^*S*^_*MAX*_	*I*^*S*^_*MIN*_/*I*^*S*^_*MAX*_	0.36 ± 0.05	0.35 ± 0.04	1.03
Ratio of *I*^*S*^_*MED*_–*I*^*S*^_*MAX*_	*I*^*S*^_*MED*_/*I*^*S*^_*MAX*_	0.77 ± 0.05	0.74 ± 0.04	1.04

^**1**^*n* = 8 except for mass (*n* = 2 for mass; the average density was obtained from mean values of volume and mass)

^**2**^ From Sim et al. 2008 and Sim et al. 2013 except for mass and average density (mass of a stapes was measured; the average density was calculated from the mean values of volume and the measured mass)

The values of the principal moments of inertia of the sheep stapes were 22–23% of those of the human stapes, and the relative ratios between the three principal moments of inertia were similar for the human and sheep stapes. The principal axes with the minimum (red lines in Fig. 2c) and maximum (black lines in Fig. 2c) moments of inertia were approximately aligned in the lateral–medial direction and in the superior–inferior direction, respectively, for both the human and sheep.

### Lever ratio and inertial properties of the malleus–incus complex

The principal axes of the malleus–incus complex in sheep and human are shown in (Fig. 2d), and the corresponding principal moments of inertia are shown in (Table 4). The principal axis for the minimum moment of inertia (red lines) is aligned close to the superior–inferior direction in the human and between the anterior–posterior direction and the superior–inferior direction in the sheep. The principal axis for the maximum moment of inertia (black lines) is aligned close to the lateral-medial direction for both sheep and human.

**Table 4:** Dimensions and inertial properties of the malleus–incus complex in sheep and humans

Description	Symbol	^1^Sheep	^2^Ref. human	Sheep/human
Length from hinge-like rotational axis to umbo	*L*_*1*_ (mm)	4.16 ± 0.26	–	–
Length from hinge-like rotational axis to ISJ	*L*_*2*_ (mm)	1.69 ± 0.09	–	–
Ratio of *L*_*1*_–*L*_*2*_ (lever ratio)	*L*_*1*_/*L*_*2*_	2.47 ± 0.28	1.25	1.98
Volume	*V*^*MI*^ (mm^3^)	9.56	27.6 ± 4.5	0.35
Mass	*m*^*MI*^ (mg)	21.1	62.2 ± 10.1	0.34
Average density	*ρ*^*MI*^ (mg/ mm^3^)	2.21	2.26	0.98
Minimum principal moment of inertia	*I*^*MI*^_*MIN*_ (mg·mm^2^)	13.2 ± 3.4	124.1 ± 31.0	0.11
Medium principal moment of inertia	*I*^*MI*^_*MED*_ (mg·mm^2^)	67.8 ± 25.4	171.0 ± 30.7	0.40
Maximum principal moment of inertia	*I*^*MI*^_*MAX*_ (mg·mm^2^)	73.6 ± 26.7	249.0 ± 52.6	0.30
Ratio of *I*^*MI*^_*MIN*_–*I*^*MI*^_*MAX*_	*I*^*MI*^_*MIN*_/*I*^*MI*^_*MAX*_	0.18 ± 0.02	0.50 ± 0.02	0.36
Ratio of *I*^*MI*^_*MED*_–*I*^*MI*^_*MAX*_	*I*^*MI*^_*MED*_/*I*^*MI*^_*MAX*_	0.92 ± 0.01	0.69 ± 0.04	1.33
Moment of inertia along hinge-like rotational axis	*I*^*MI*^_*AXIS*_ (mg·mm^2^)	46.2 ± 17.7	171.5 ± 17.6	0.27
Ratio of *I*^*MI*^_*AXIS*_–*I*^*MI*^_*MED*_	*I*^*MI*^_*AXIS*_/*I*^*MI*^_*MED*_	0.67 ± 0.00	1.02 ± 0.10	0.66
^**3**^Moment of inertia in anterior–posterior axis	*I*^*MI*^_*AP*_ (mg·mm^2^)	58.8	153.6	0.38
^**3**^Moment of inertia in superior–inferior axis	*I*^*MI*^_*SI*_ (mg·mm^2^)	52.1	111.9	0.47
^**3**^Moment of inertia in lateral-medial axis	*I*^*MI*^_*LM*_ (mg·mm^2^)	85.9	211.7	0.47
^**3**^Ratio of *I*^*MI*^_*AP*_–*I*^*MI*^_*AXIS*_	*I*^*MI*^_*AP*_/*I*^*MI*^_*AXIS*_	1.00	0.91	1.11
^**3**^Ratio of *I*^*MI*^_*SI*_–*I*^*MI*^_*AXIS*_	*I*^*MI*^_*SI*_/*I*^*MI*^_*AXIS*_	0.89	0.66	1.35
^**3**^Ratio of *I*^*MI*^_*LM*_–*I*^*MI*^_*AXIS*_	*I*^*MI*^_*LM*_ /*I*^*MS*^_*AXIS*_	1.46	1.25	1.17
Distance between rotational axis to C.O.M	*d*^*MI*^_*AXIS-COM*_ (mm)	0.60 ± 0.04	0.83 ± 0.11	0.73

^**1**^*n* = 2 for lever ratio and principal moments of inertia, and the volume, mass, and density were obtained from the average values of the malleus and incus in Tables 1, 2

^**2**^Data from Sim et al. 2007 (*n* = 3) and one additional measurement (*n* = 4 in total), except for the lever ratio (the lever ratio from Rosowski et al. 1996 and Nummela and Sánchez-Villagra, 2006)

^**3**^The moments of inertia in the anatomical frame and related ratios were obtained from only one sample with an intact middle ear in each of the sheep and human

The malleus–incus complex of the sheep had a volume and mass of 34–35% of those in the human, and all of the minimum, medium, and maximum principal moments of inertia in the sheep were smaller than the corresponding principal moments of inertia in the human. The ratio of the minimum principal moment of inertia to the maximum principal moment of inertia (*I*^*MI*^_*MIN*_/*I*^*MI*^_*MAX*_) was 0.51 ± 0.01 in the human and only 0.18 ± 0.02 in the sheep. The medium principal moment of inertia was almost the same as the maximum principal moment of inertia in the sheep (*I*^*MI*^_*MED*_/*I*^*MI*^_*MAX*_ = 0.92 ± 0.01), but was not in the human (*I*^*MI*^_*MED*_/*I*^*MI*^_*MAX*_ = 0.69 ± 0.04). The moment of inertia along the hinge-like rotational axis (*I*^*MI*^_*AXIS*_) was relatively small in the sheep compared to the corresponding moment of inertia in the human. The ratio of the moment of inertia along the hinge-like rotational axis to the medium principal moment of inertia (*I*^*MI*^_*AXIS*_/*I*^*MI*^_*MED*_) was 0.67 ± 0.00 in the sheep and 1.02 ± 0.10 in the human, indicating a relatively large moment of inertia along the hinge-like rotational axis in the human. The distance between the hinge-like rotational axis and the ossicular C.O.M. was 0.60 ± 0.04 mm in the sheep and 0.83 ± 0.11 mm in the human. The ratio of the distance of the sheep to the distance of the human was 0.73. Considering other length ratio between the sheep and the human (e.g., the ratio of the long length of the footplate between the sheep and the human was 0.72 in Table 3), the distance between the hinge-like rotational axis and the ossicular C.O.M. can be considered to be similar for the sheep and the human.

The malleus–incus complex of the sheep has a large lever ratio (*L*_*1*_/*L*_*2*_ ≈ 2.47) compared to that of the human (*L*_*1*_/*L*_*2*_ ≈ 1.25) by a factor of 1.98.

### Inertial properties of the middle-ear ossicular chain in the global intrinsic frame

**Fig. 3:**
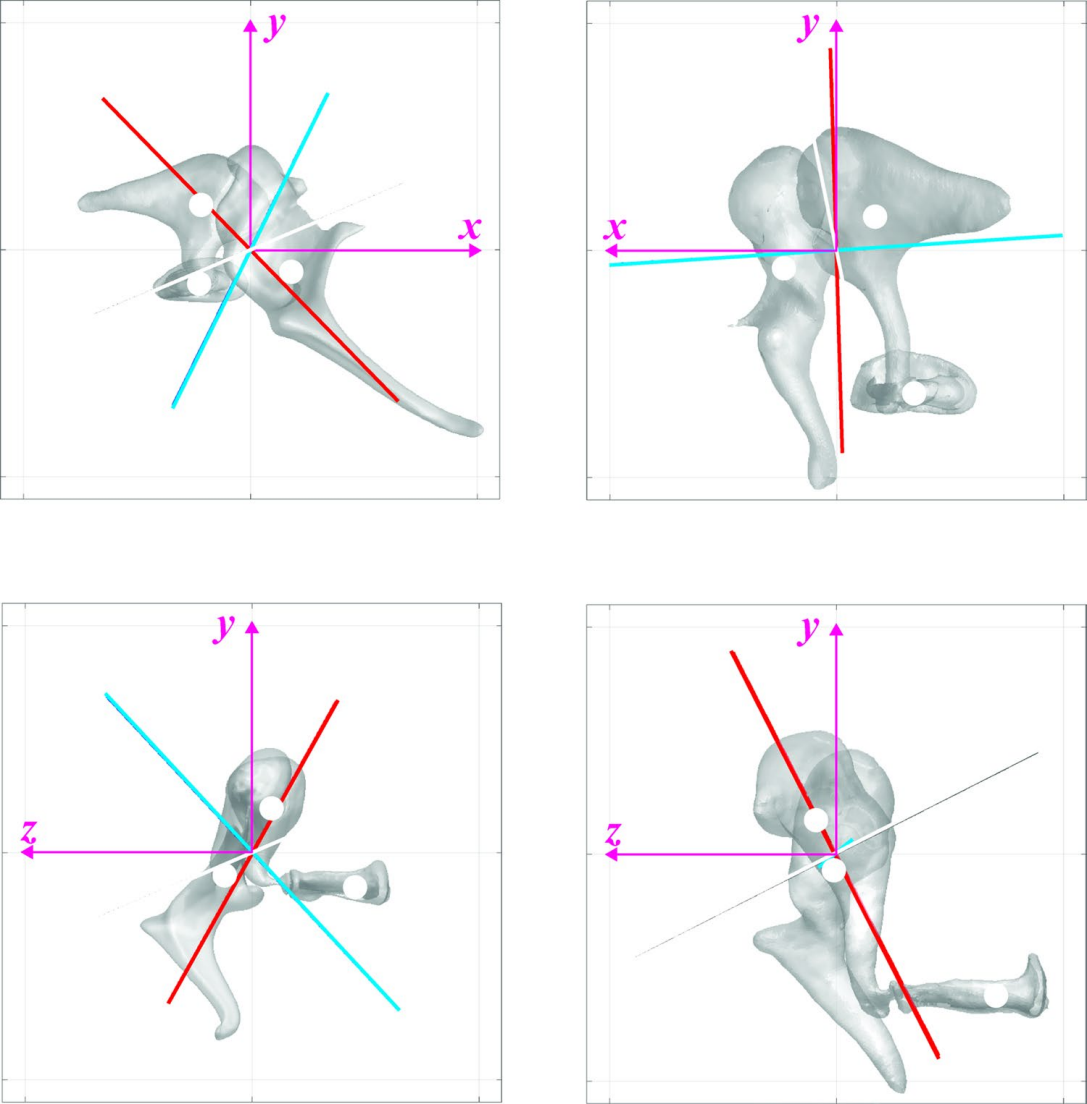
The middle-ear ossicles in the global anatomical intrinsic frame (*xyz* frame) based on the stapes footplate, of the sheep (**a**, right ear) and human (**b**, left ear). The positive *x-*, *y-*, and *z*-directions correspond to the anterior (corresponds to the rostral for human and sheep), superior (corresponds to the dorsal for sheep), and lateral directions, respectively. The red, blue, and black lines indicate the principal axes with the minimum, medium, and maximum principal moments of inertia, respectively. The origin (also the intersection of the principal axes) in each figure corresponds to the center of the mass (C.O.M.) of the entire ossicular chain, and the checkered circles indicate the center of mass (C.O.M.) of the malleus, incus, and stapes

The entire middle-ear ossicular chain in the sheep had a volume and mass of 35–36% of those in the human (Table 5). The ratio of the minimum to the maximum moments of the inertia (*I*^*MIS*^_*MIN*_/*I*^*MIS*^_*MAX*_) was much smaller in the sheep (0.28 in the sheep versus 0.51 in the human), indicating that the minimum moment of inertia in the sheep is relatively small compared to that of human. While the principal axis with the minimum moment of inertia was aligned along the superior–inferior direction in the human (red lines in Fig. 3b), the axis was aligned between the anterior–posterior direction and the superior–inferior direction in the sheep (red lines in Fig. 3a). The axis with the maximum moment of inertia was aligned between the anterior–posterior direction and the lateral-medial direction in the sheep (black lines in Fig. 3a), and between the superior–inferior direction and the lateral-medial direction in the human (black lines in Fig. 3b).

**Table 5:** Inertial properties of the entire middle-ear ossicular chain in sheep and humans

Description	Symbol	^1^Sheep	^2^Ref. human	Sheep/human
Volume	*V*^*MIS*^ (mm^3^)	10.4	28.9	0.36
Mass	*m*^*MIS*^ (mg)	22.9	65.4	0.35
Average density	*ρ*^*MIS*^ (mg/ mm^3^)	2.20	2.26	0.97
Minimum principal moment of inertia	*I*^*MIS*^_*MIN*_ (mg·mm^2^)	30.1	144.9	0.18
Medium principal moment of inertia	*I*^*MIS*^_*MED*_ (mg·mm^2^)	95.8	240.7	0.38
Maximum principal moment of inertia	*I*^*MIS*^_*MAX*_ (mg·mm^2^)	104.2	292.1	0.33
Ratio of *I*^*MIS*^_*MIN*_–*I*^*MIS*^_*MAX*_	*I*^*MIS*^_*MIN*_/*I*^*MIS*^_*MAX*_	0.29	0.50	0.58
Ratio of *I*^*MIS*^_*MED*_–*I*^*MIS*^_*MAX*_	*I*^*MIS*^_*MED*_/*I*^*MIS*^_*MAX*_	0.92	0.82	1.11
Principal direction with minimum PMOI	***n***^*MIS*^_*MIN*_	(0.65, −0.67, 0.37)	(−0.03, −0.89, -0.45)	–
Principal direction with middle PMOI	***n***^*MIS*^_*MID*_	(−0.34, −0.69, −0.64)	(0.99, −0.06, 0.07)	–
Principal direction with maximum PMOI	***n***^*MIS*^_*MAX*_	(0.68, 0.29, −0.67)	(−0.09, −0.45, 0.89)	–
Moment of inertia along hinge-like rotational axis	*I*^*MIS*^_*AXIS*_ (mg·mm^2^)	72.1	266.9	0.27
Moment of inertia in anterior–posterior axis	*I*^*MIS*^_*AP*_ (mg·mm^2^)	72.1	241.1	0.30
Moment of inertia in superior–inferior axis	*I*^*MIS*^_*SI*_ (mg·mm^2^)	67.4	175.1	0.39
Moment of inertia in lateral-medial axis	*I*^*MIS*^_*LM*_ (mg·mm^2^)	90.6	261.5	0.35
Ratio of *I*^*MIS*^_*AP*_ to *I*^*MIS*^_*AXIS*_	*I*^*MIS*^_*AP*_/*I*^*MIS*^_*AXIS*_	1.00	0.90	1.11
Ratio of *I*^*MIS*^_*SI*_ to *I*^*MIS*^_*AXIS*_	*I*^*MIS*^_*SI*_/*I*^*MIS*^_*AXIS*_	0.93	0.66	1.42
Ratio of *I*^*MIS*^_*LM*_ to *I*^*MIS*^_*AXIS*_	*I*^*MIS*^_*LM*_ /*I*^*MIS*^_*AXIS*_	1.26	0.98	1.28
Distance between rotational axis to C.O.M	*d*^*MIS*^_*AXIS-COM*_ (mm)	0.66	0.86	0.77
Center of mass of malleus in intrinsic frame	*CM*^*M*^ (mm)	(0.87, −0.50, 0.60)	(1.16, −0.38, 0.02)	–
Center of mass of incus in intrinsic frame	*CM*^*I*^ (mm)	(−1.11, 0.96, −0.43)	(−0.84, 0.71, 0.40)	–
Center of mass of stapes in intrinsic frame	*CM*^*S*^ (mm)	(−1.14, −0.76, −2.25)	(−1.70, −3.14, −3.53)	–
Center of mass of stapes in intrinsic frame	*CM*^*MI*^ (mm)	(0.10, 0.07, 0.20)	(0.11, 0.20, 0.22)	

^1^From one sample with an intact middle ear except for volume, mass, and density (the volume, mass, and density were obtained from the average values of the malleus and incus in Tables 1, 2, and 3)

^2^From one sample with the intact middle ear except for volume, mass, and density (the volume, mass, and density were obtained from the average values of the malleus and incus in Tables 1, 2, and 3)

The moments of inertia in the global intrinsic anatomical frame were calculated for the entire middle-ear ossicular chain (*I*^*MIS*^_*AP*_, *I*^*MIS*^_*SI*_, and *I*^*MIS*^_*LM*_ in Table 5). The moment of inertia along the hinge-like rotational axis was calculated as well. The results indicated that the moments of inertia in the superior–inferior direction and in the lateral-medial direction relative to the moment of inertia in the hinge-like rotational axis were larger in the sheep (both *I*^*MIS*^_*SI*_/*I*^*MIS*^_*AXIS*_ and *I*^*MIS*^_*LM*_/*I*^*MIS*^_*AXIS*_ were larger in the sheep) than in human. The moment inertia along the hinge-like rotational axis was similar to the moment of inertia along the anterior–posterior axis in both species (*I*^*MIS*^_*AP*_/*I*^*MIS*^_*AXIS*_ was 1.00 in the sheep and 0.90 in the human). The distance between the hinge-like rotational axis and the ossicular C.O.M was 0.66 mm in the sheep and 0.86 mm in the human.

## Discussion

### Volume of the middle-ear ossicles

The total volume of the three middle-ear bones was approximately 10.4 mm^3^ in the sheep and 28.9 mm^3^ in the human. Therefore, the overall volume of the middle-ear bones in the human is larger by a factor of 2.8 than in the sheep. The volume ratios of the malleus, incus, and stapes to the total volume are 55%, 37%, and 8% in the sheep, and 44%, 51%, and 5% in the human. Considering the relative volume ratios, the sheep middle ear has a relatively large malleus and stapes, and a relatively small incus, compared to the human middle ear. According to an investigation of ossicular mass of mammal middle ears by Nummela (1995), the larger size of the incus, like the human in this study, was found mainly in species belonging to Primates and Pinnipedia (seals), and in two species (*Camelus bactrianus* and *Bos taurus*) belonging to Artiodactyla. By contrast, the malleus is heavier than the incus in other mammals, which belong to Eulipotyphla (the order Insectivora has been abandoned. Eurasian hedgehog, Desert hedgehog and Common mole in Nummela (1995) belong to Eulipotyphla now), Chiroptera, Lagomorpha, Rodentia, Carnivora, Proboscidea, Perissodactyla, and Artiodactyla (except for *Camelus bactrianus* and *Bos taurus*).

### Anatomy of the malleus

The shape and the alignment of the sheep malleus have two major differences from that of the human malleus. First, the sheep malleus has a slender shape. While the volume of the sheep malleus is less than half the volume of the human malleus, the lengths of the sheep malleus are almost equivalent. Second, while the malleus handle of the human is approximately along the superior–inferior direction, the malleus handle of the sheep is tilted into the anterior–posterior direction, making an angle of about 45° between the longitudinal direction of the malleus handle and the anterior–posterior direction (Fig. 3). Since the anatomical directions in this article were defined based on orientation of the stapes (see “Materials and methods”), tilting of the malleus handle relative to the head may be different. However, considering the fact that the orientation of the incus is similar in both species, with the anatomical orientation defined in this article (Fig. 3), the sheep malleus handle is tilted by approximately 45° compared to orientation of the human malleus handle, when the middle-ear ossicles of both species are aligned with orientations of the incus and the stapes. The slender shape contributes to making a large lever ratio for the hinge-like rotational motion about an axis along the anterior–posterior direction. The alignment of the sheep malleus handle, deviating from the superior–inferior direction, generates a relatively large moment of inertia along the superior–inferior direction, compared to the alignment of the malleus handle along the superior–inferior direction in the human. Comparing the two alignments, the principal axis with the minimum moment of inertia (red lines in Fig. 2) is close to the superior–inferior direction in the human malleus, and deviates from the superior–inferior direction in the sheep malleus. From the viewpoint of mechanics, this indicates that rotation of the human malleus about the superior–inferior direction is prone to being generated at high frequencies because rotational inertia about the superior–inferior direction is close to the minimum. Such a rotational motion of the human malleus at high frequencies, which is denoted as “torsional motion” of the malleus in the literature, was predicted by Puria and Steele (2010) and was observed in measurements in the human above 3 kHz by Dobrev et al. (2016). Considering the fact that the principal axis with the minimum moment of inertia deviates from the superior–inferior direction in the sheep malleus, it is expected that the sheep malleus would not have considerable “torsional” motion at high frequencies. If only the moment of inertia of the malleus is considered, and it is assumed that the malleus has a loose connection to the incus, the sheep malleus may have rotational motion along the along the axis through the malleus handle, which has the anteroinferior orientation, at high frequencies. However, motion of the malleus at high frequencies is determined by flexibility of the IMJ as well. According to previous works (Willi et al. 2002; Puria and Steele 2010; Gerig et al. 2015; Dobrev et al. 2016), in mammals with deformable IMJ (e.g., human and cat), connection of the malleus to the incus can be considered to be loose allowing relative motion between the malleus and the incus only at high frequencies above the first natural (resonance) frequencies. As for species with a naturally fixed IMJ, Puria and Steele (2010) hypothesized that motion of the malleus–incus complex would maintain hinge-like rotational motion even at high frequencies. Therefore, to predict motion of the sheep malleus at high frequencies, flexibility of the IMJ needs to be investigated.

### Anatomy of the incus

In addition to its small volume relative to the human incus, the sheep incus has a relatively short length of the long process. This shape reduces the length of the lever arm of the incus (*L*_2_) in the hinge-like rotational motion about an axis along the anterior–posterior direction and makes the moment of the inertia along anterior–posterior direction close to the minimum (because the principal axis with minimum moment of inertia (red line) is aligned approximately along the anterior–posterior direction for the sheep incus in Fig. 2). Therefore, the shape of the sheep incus provides a large lever ratio and a small rotational inertia for the hinge-like rotational motion along the anterior–posterior direction.

### Anatomy of the stapes

Though the ratio of the height to the footplate size (*h/d*_*FTproj_eq*_) is larger in the human stapes, the position of the center of mass relative to the total height (*h*_*c*_*/h*) is almost the same for the two. This is due to the relatively large stapes head in the sheep. With the large size of the head, the articular face of the incudostapedial joint (ISJ) becomes large (*A*_*ISJ*_ = 0.42 ± 0.05 mm^2^ in the sheep and *A*_*ISJ*_ = 0.24 ± 0.07 mm^2^ in the human in Table 2). The large area of the articular face of the ISJ in the sheep may make a strong and reliable connection between the incus and stapes, which may make the relative movement between the incus and the stapes smaller than that in the human.

### Contribution of the middle-ear anatomy to the hinge-like rotational motion

Acoustic impedance is defined as the ratio of the sound pressure in the medium to the volume flow rate by the vibrating structure. While the acoustic impedance of the cochlea varies with frequency (Merchant et al. 1996; Puria et al. 1997; Aibara et al. 2001; Nakajima et al. 2009; Péus et al. 2017), it is much larger through all frequencies than the acoustic impedance of the air. The specific acoustic impedance (multiplication of the acoustic impedance and the cross-sectional area of the vibrating structure) of the human cochlea by Zwislocki (1965) was 56 kPa s m^−1^, which is much larger than the acoustic impedance of the air (≈ 420 Pa s m^−1^). For sound energy to be transferred to the cochlear fluid via the middle ear overcoming the large impedance difference, pressure gain through the middle ear is necessary. The expected middle-ear pressure gain by hinge-like rotational motion can be calculated by multiplying the area ratio between the pars tensa of the tympanic membrane and oval window by the lever ratio of the malleus–incus complex. The lever ratio of the sheep obtained in this study is larger than the lever ratio of the human by a factor of 1.98 (Table 4), resulting in a smaller piston-like motion of the stapes relative to the motion of the umbo in the sheep. With the lever ratio obtained in this study, the motion at the incus lenticular process is expected to be smaller than the motion at the umbo, by 1.94 dB in the human and by 7.85 dB in the sheep. According to previous studies, the motion at the lenticular process of the incus is smaller than the motion at the umbo, by 3 dB at frequencies below 1.5 kHz in the human (Dobrev et al. 2016), and by 10 dB at frequencies below 2 kHz in the sheep (Peus et al. 2017). The area ratio between the tympanic membrane and oval window can be calculated from the projected areas of the footplate in (Table 3) and the areas of the tympanic membrane reported in the literature. With 68.3 mm^2^ and 44.2 mm^2^ for the areas of the tympanic membrane of the human and sheep (Nummela 1995), respectively, the resulting area ratio is 23.9 for the human and 30.5 for the sheep. Considering that the area ratio is larger in the sheep, the larger lever ratio is not presumed to be for compensation of the area ratio. With the area ratios and the lever ratios (2.47 for the sheep and 1.25 for the human) obtained in this study, the expected middle-ear pressure gain is approximately 75 for the sheep and approximately 30 for the human.

Dallos (1973) explained efficiency of impedance matching between the air and the cochlear fluid using a simple model for rotational motion along the anatomical axis. According to the work, transfer of the sound energy from air to the middle ear can be enlarged as the specific acoustic impedance of the tympanic membrane (*Z*_TM_) approaches to the specific acoustic impedance of the air (*Z*_a_ ≈ 420 Pa s m^−1^). In the work, the specific acoustic impedance of the tympanic membrane was calculated using an ideal transformer theory [Eq. (3.18) in the article], with a value of 56 kPa s m^−1^ for the specific acoustic impedance of the cochlea (*Z*_*cochlea*_). If the lever ratios and area ratios of the sheep and the human are applied to the formula, 301 Pa s m^−1^ and 1500 Pa s m^−1^ are obtained for the specific acoustic impedances of the tympanic membrane of the sheep and the human, respectively. Then the proportion of incident sound energy that is transferred from air into the middle [calculated by Eq. (3.14) in the article] becomes 97% for the sheep and 68% for the human. Mason (2016) critically discussed about limitation of such an approach using a simple model with the ideal transformer theory. However, considering the much large proportion of sound energy transferred to the middle in the sheep, the lever ratio and the area ratio of the sheep are expected to be more efficient in transferring sound energy into the middle ear with hinge-like rotational motion than the lever ratio and the area ratio of the human.

The lever ratio and area ratio have been investigated on 23 marsupial species belonging to Didelphimorphia, Dasyuromorphia, and Diprotodontia by Nummela and Sánchez-Villagra (2006). All species in their study had larger lever ratios and larger area ratios than the human, resulting in a larger expected middle-ear pressure gain (ranging from 57.6 to 140.9) than the expected pressure gain of the human (≈ 30). In a work by Mason (2001), morphometry of the middle ear was compared between non-fossorial and fossorial mammals. According to the study, non-fossorial mammals have the larger mean area ratio and mean lever ratio than fossorial mammals, indicating that the area ratio and lever ratio might become larger evolutionarily. The mean area ratio (28.27 from 102 species) and the mean lever ratio (2.24 from 83 species) of the non-fossorial mammals are close to the corresponding ratios of the sheep rather than the human. Only two out of 83 non-fossorial species show the lever ratio smaller than the lever ratio of the human (the lever ratios of *Didelphis marsupialis* and *Solenodon paradoxus* are 1.19 and 1.20, respectively). The expected pressure gains of the investigated species can be obtained from the area ratios and lever ratios provided by the study (e.g., 118 for the chinchilla, 72 for the gerbil, 85 for the guinea pig, and 79 for the cat).

The middle-ear pressure was compared between the sheep and the human only for the hinge-like rotational motion along the fixed rotational axis in this article, assuming that the malleus–incus complex behaves like a rigid body without relative motion between the malleus and the incus. While certain rodents such as guinea pigs have a fused incudomalleal joint (IMJ), nearly all other mammals have a deformable IMJ (Puria and Steele 2010; Mason 2013, 2015; Mason and Farr 2013). Previous studies suggested that the malleus–incus complex of the human behaves like a rigid body up to the first natural frequency, and the malleus and incus have relative motion above the natural frequency. Dobrev et al. (2016) showed that the hinge-like rotational motion malleus–incus complex is dominant up the natural frequency (~ 1.5 kHz) without considerable relative motion between the malleus and the incus up to the natural frequency. Willi et al. (2002) observed considerable relative motion between the malleus and the incus in rotational motion along the anterior–posterior axis only above the natural frequency. Such relative motion was observed in the isolated malleus–incus complex by Sim et al. (2004) as well. Gerig et al. (2015) showed that 3D motion of the stapes is almost the same up to the natural frequency for flexible and fixed conditions of the IMJ, indirectly indicating that the incudomalleal joint is almost fixed up to the natural frequency. The hinge-like rotational axis has been known to be fixed at low frequency and vary at high frequencies. Dobrev et al. (2016) showed that in the human, motion at the incus lenticular process and motion at the umbo have similar magnitude ratios with similar phases up to the natural frequency. Decraemer et al. (2014) showed in his measurements with gerbils, which have a fused IMJ, that the rotational axis is close the anatomically determined axis at low frequencies, and is deviated from the anatomically determined axis at higher frequencies. While the natural frequency of the sheep middle ear is known to be near 4.8 kHz (Péus et al. 2017), flexibility of the IMJ in the sheep has not been known. However, with previous studies considered, regardless of flexibility of the IMJ, it is expected that dominant motion of the sheep malleus–incus complex is hinge-like rotational up the natural frequency without considerable relative motion between the malleus and the incus.

The malleus–incus complex of the sheep has a relatively small moment of inertia along the hinge-like rotational axis compared to the corresponding moment of inertia in the human (*I*^*MI*^_*AXIS*_/*I*^*MI*^_*MED*_ = 0.67 ± 0.003 in the sheep and *I*^*MI*^_*AXIS*_/*I*^*MI*^_*MED*_ = 1.02 ± 0.10 in the human in Table 4). It means that mass distribution of the sheep malleus–incus complex impedes the hinge-like rotational motion less. Mass distribution of the malleus–incus complex in the human tends to make a relatively small moment of inertia along the superior–inferior axis (*I*^*MI*^_*SI*_/*I*^*MI*^_*AXIS*_ = 0.89 for the sheep and *I*^*MI*^_*SI*_/*I*^*MI*^_*AXIS*_ = 0.66 for the human in Table 4). With such moments of inertia, it is expected that the human malleus–incus complex would have a larger rotational motion along the superior–inferior direction at high frequencies. It is known that motion of the human malleus–incus complex has such motion at frequencies above 1.5 kHz (Dobrev et al. 2016). Consequently, such a motion would likely be smaller in the sheep middle ear, but measurement of the three-dimensional motion of the sheep middle ear has not been reported.

It was also observed that the cross section of the malleus handle in the sheep has a rectangular shape with the long side along the lateral-medial direction in comparison to the circular shape in human. The rectangular cross-sectional shape of the sheep malleus handle is optimal for providing strength against the bending caused by the hinge-like motion of the middle ear about an axis along the anterior–posterior direction; whereas, the circular cross-sectional shape of the human malleus handle provides isotropic strength.

The large lever ratio, the large area ratio of the tympanic membrane relative to the oval window, the relatively small moment of inertia along the anterior–posterior direction, and the rectangular cross-sectional shape of the malleus handle in the sheep middle ear suggest that the sheep middle ear is designed more optimally for the hinge-like motion of the middle ear ossicular chain about an axis along the anterior–posterior direction with a larger middle-ear pressure gain.

### Evolutionary aspects

Fleischer (1978) used terminology of “freely mobile” and “microtype” to explain two main streams in evolutionary radiation of the malleus–incus complex and the tympanic membrane in terrestrial mammals. The freely mobile malleus–incus complex, which is generally found in mammals with ears of a medium or large size including humans, has a malleus with a large head and a relatively large incus. The malleus in this category is freely mobile due to its loose connection to the tympanic bone via a relatively short anterior process. On the contrary, the microtype malleus–incus complex, which is closer to the ancestral type than the freely mobile malleus–incus complex, is found in mammals with small-sized ears such as bats and mice. The middle ear in this category has a relatively small incus and the malleus has an orbicular apophysis and a wide transversal lamina. The malleus in this category has a firm connection to the tympanic bone via an elongated anterior process. Another important difference between the freely mobile and microtype malleus–incus complexes is in orientation and location of the axis of hinge-like rotational motion. In the work by Fleischer, the anatomical axis, which passes through the anterior process of the malleus and the tip of the short process of the incus where the ossicles are attached to the skull, was approximated as the rotational axis of hinge-like rotational motion (the same as approximation of the rotational axis in this article). While the anatomical axis in the freely mobile malleus–incus complex is approximately perpendicular to the malleus handle and is located near the ossicular center of mass, the anatomical axis in the microtype malleus–incus complex takes a small angle to the malleus handle (or even almost parallel to the malleus handle in some cases) and is located further from the ossicular center of mass. Due to the further distance of the rotational axis from the ossicular center of mass, the microtype malleus–incus complex has a large moment of inertia along the rotational axis (see Materials and methods), and thus is not optimal for the hinge-like rotational motion.

While the human malleus–incus complex apparently belongs to the freely mobile malleus–incus complex, the sheep malleus–incus complex has several features of the freely mobile type and several features of the microtype. The sheep has a malleus with a transversal lamina and its oblique orientation relative the rotational axis and a relatively small incus, which belong to the characteristics of the microtype malleus–incus complex. On the other hand, the sheep malleus does not possess an orbicular apophysis and the rotational axis is close to the ossicular center of mass, which belong to the features of the freely mobile malleus–incus complex. The moment of inertia along the rotational axis in the sheep is relatively small compared to one in the human. Considering a fact that the sheep malleus–incus complex has characteristics of both the freely mobile and microtype malleus–incus complexes in part, the sheep middle ear can be categorized into “transitional type”, which is a term for a hybrid between the ancestral and freely mobile types in the work by Fleischer (1978). Such a transitional type of the malleus–incus complex is observed in species of rodents as well. In a work by Lavender et al. (2011), morphology and inertial properties of seven species in rodents were examined. Among the seven species, five species of *Rattus*, *Cricetulus*, two *Phodopus* species (*sungorus* and *roborovskii*), and *Mesocricetus* could be considered to belong to the transitional type. These the five species have a relatively small incus compared to the human. The ossicular center of mass is located relatively near the rotational axis in two *Phodopus* species and the *Mesocricetus*, like the sheep. While the *Rattus* and the *Cricetulus* have an extended transverse lamina with a small orbicular apophysis, the *Mesocricetus* has a non-extended transverse lamina without an orbicular apophysis, like the sheep. When orientations of the malleus handle and the principal axis with the minimum moment of inertia relative to the rotational axis are considered, the *Cricetulus* and the *Phodopus roborovskii* show orientations similar to the corresponding orientations in the sheep. The five species and the sheep show diversity in the transitional type between the freely mobile and microtype malleus–incus complexes.

It has been known that mammals of the freely mobile type generally have better low-frequency hearing than mammals of the microtype (Fleischer 1978; Lavender et al. 2011). The lower limit of the audible hearing range with 60 dB SPL is 125 Hz for the sheep and 29 Hz for the human (West 1984). Considering the fact that the sheep malleus–incus complex provide better conditions for the middle-ear pressure gain by the hinge-like rotational motion than the human malleus–incus complex, it is presumed that the lower hearing limit is not determined by the middle-ear pressure gain by the hinge-like rotational motion.

### Contribution of the middle-ear anatomy to the rocking-like motion of the stapes

**Fig. 4:**
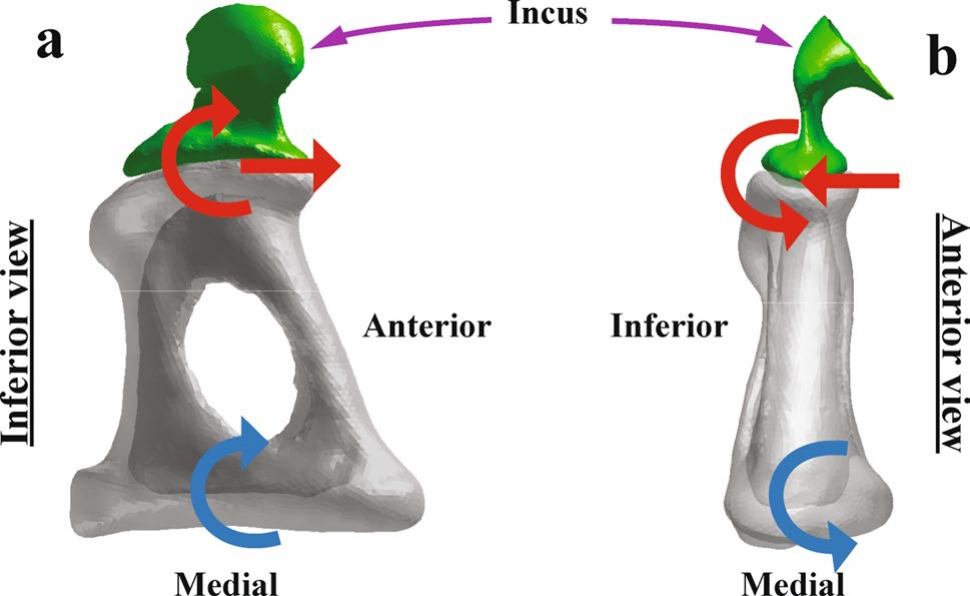
The rocking-like motions (blue) about the short (**a**) and long (**b**) axes of the stapes footplate, and the resulting motions (red) at the stapes head

It has been reported that the three-dimensional motion of the human stapes footplate is composed of both rocking-like and piston-like motions (e.g., Hato et al. 2003; Sim et al. 2010). According to one theory of dynamics, while the piston-like motion of the stapes footplate generates only translational motion of the stapes head, the rocking-like motion of the stapes footplate (blue in Fig. 4) generates rotational motion as well as translational motion of the stapes head (red in Fig. 4). That is, the rocking-like motion about the short axis of the footplate generates a translation of the stapes head in the anterior–posterior direction and a rotation about an axis along the superior–inferior direction (Fig. 4a), and the rocking-like motion about the long axis of the footplate generates a translation of the stapes head in the superior–inferior direction and a rotation about an axis along the anterior–posterior direction (Fig. 4b). The incudostapedial joint (ISJ) in the sheep has a larger area of the articular face (*A*_*ISJ*_ in Table 2) by a factor of 1.75 than the ISJ in the human whereas the length of the incus lenticular process (*L*_*LP*_ in Table 2) and the height of the stapes (*h* in Table 3) in the sheep are smaller by factors of 0.40 and 0.64, respectively, than the corresponding dimensional lengths in the human. More specifically, the articular face has a long length along the anterior–posterior direction in the sheep. Further, while the long process of the human incus has a portion with a very small cross-sectional area near the lenticular process (pedicle, Funnell et al. 2005), the long process in the sheep incus still has a large cross-sectional area near the lenticular process. Considering the shape and area of the articular faces at the ISJ and the cross section of the long process near the lenticular process, the rotational motion of the stapes head around a superior–inferior axis (Fig. 4a) is presumed to be prevented in the sheep; therefore, the rocking-like motion about the short axis of the footplate is expected to be minimized as well.

### Summary and sheep middle ear as a surrogate of the human middle ear

Anatomy of the sheep middle-ear ossicular chain in comparison to the human middle-ear ossicular chain is characterized by a slenderer shape and a relatively larger moment of inertia along the superior–inferior axis of the malleus, a relatively small size and a small moment of inertia along the anterior–posterior axis of the incus, a relatively small footplate area of the stapes, and a relatively large interface area of the ISJ. The relative magnitude of the moment of inertia of the malleus–incus complex along the hinge-like rotational axis is smaller in the sheep (The ratios of *I*^*MI*^_*SI*_/*I*^*MS*^_*AXIS*_, *I*^*MS*^_*SI*_/*I*^*MI*^_*AXIS*_, and *I*^*MS*^_*LM*_/*I*^*MI*^_*AXIS*_ of the sheep are larger than the corresponding ratios of the human in Table 4). All the anatomical features of the sheep middle-ear ossicular chain support a hypothesis that the middle-ear ossicular chain of the sheep is more efficient for the hinge-like rotational motion with a larger middle-ear pressure gain. Although Puria and Steele (2010) suggested that the larger “torsional” motion of the malleus and the flexible incudomalleal joint (IMJ) of the human are for efficiency of high-frequency sound transmission at high frequencies, Gerig et al. (2015) determined that the deformable IMJ has a negative effect on middle-ear sound transmission at high frequencies. It is unclear why middle-ear pressure gain is sacrificed in the human. The work by Gerig et al. suggested that the mobility of the IMJ may exist to act as a spatial buffer for protection against high-level sound thus supporting a protection hypothesis for loss of middle ear sound transmission. The works by Hüttenbrink (1988) and Ihrle et al. (2016) observed that position change of the malleus, caused by change of quasi-static pressure, generates relative movement between the malleus and the incus. They suggested that the relative movement by the flexible IMJ reduces motions of the incus and stapes relative to motions of the malleus and thus protects the inner ear, in cases of significant static pressure difference between the ear canal and middle-ear cavity. Further, efficiency in stiffening and thus constraining motion of the middle-ear ossicular chain by the two middle-ear muscular tendons is presumed to be dependent on anatomy of the middle ear. The relation between stiffening of the middle-ear ossicular chain by the two middle-ear muscular tendons and the middle-ear anatomy is not discussed further in this article because it is more appropriately explored with a comprehensive middle-ear model.

Different criteria may be used in support of various animal models considering the purpose for the model. If surgical training or surgical access for new surgical techniques is the main purpose, then the sizes of the ossicles and the middle-ear cavity would be the main criteria. However, considering substantial differences in anatomy and functions (e.g., protection mechanism) of the middle ear between the sheep and human, surgical outcomes would be difficult to predict directly from measurements using the sheep middle ear. To assess surgical outcomes from measurements using the sheep middle ear, middle-ear mechanics of the sheep would need to be comprehensively understood in comparison with middle-ear mechanics of the human. This study provides anatomical information of the sheep middle ear, which may be used for construction of comprehensive mechanical models of the sheep middle ear for better understanding of middle-ear mechanics of the sheep.

## Conclusion

This study provides quantitative data on the characteristic lengths, sizes, and inertial properties of sheep middle-ear bones, which are necessary for creating a mathematical model (multi-body model or finite element model) of the sheep middle ear. Morphometry of the soft tissues in the middle ear, which is also necessary for the mathematical model, was not included in this study and is a topic for future investigation.

Anatomical features of the sheep middle-ear bones, which are distinguishable from anatomy of the human middle-ear bones, provide better conditions for the hinge-like rotational motion of the ossicular chain along the anterior–posterior axis with a large middle-ear pressure gain.

Although the sheep middle ear can be used for training of middle-ear surgeries due to its similar size to the human middle ear, the substantial differences in middle-ear anatomy make predictions of surgical outcomes difficult to assess directly.

## Abbreviations

3D: Three dimensional
C.O.M: Center of mass
CT: Computed tomography
FT: Stapes footplate
IMJ: Incudomalleal joint
ISJ: Incudostapedial joint
MI: Malleus–incus complex consisting of the malleus and incus
MIS: Intact middle-ear ossicular chain consisting of the malleus, incus, and stapes
PMOI: Principal moments of inertia
STL: Standard tessellation language
